# Integrated genomic analysis of EDNRB common and rare variants in Hirschsprung disease

**DOI:** 10.1016/j.gendis.2025.101595

**Published:** 2025-03-04

**Authors:** Xiaoyu Zuo, Jinglu Zhao, Zipeng Liu, Yuxiao Yao, Dingyang Li, Xiaogang Xu, Huimin Xia, Lihua Huang, Jixiao Zeng, Yan Zhang

**Affiliations:** a Provincial Key Laboratory of Research in Structure Birth Defect Disease and Department of Pediatric Surgery, Guangzhou Women and Children's Medical Center, Guangzhou Medical University, Guangzhou, Guangdong 510623, China; b The Fifth Affiliated Hospital of Guangzhou Medical University, Guangzhou, Guangdong 510700, China; c The Third Affiliated Hospital of Zhengzhou University, Zhengzhou, Henan 450052, China; d Warshel Institute for Computational Biology, The Chinese University of Hong Kong, Shenzhen, Guangdong 518172, China; e School of Medicine, The Chinese University of Hong Kong, Shenzhen, Guangdong 518172, China

Hirschsprung disease (HSCR) is a congenital intestinal motility disorder characterized by absent enteric ganglia in the distal intestine.^1^ While HSCR is primarily a genetic disorder, its inheritance pattern is complex and remains largely unexplored.^2^ Current genetic screening focuses on rare variants of several essential genes (*e.g.*, *RET*, *EDNRB*, *GDNF*, *SOX10*), which unfortunately could explain only a limited portion of genetic heritability.^3^ The interplay between common and rare variants may significantly impact disease risk.^4^ Endothelin receptor type B (*EDNRB*) is a well-established HSCR contributor^5^ and was previously considered to explain about 5%–10% of HSCR children. While rare coding *EDNRB* variants are associated with HSCR, the contribution of common variants and their interaction with rare variants remains unclear. To address this, we conducted a large-scale genetic study involving 553 HSCR participants and 2075 controls from southern Chinese children, employing single nucleotide polymorphism (SNP)-array genotyping, exome sequencing, and genome sequencing (Fig. 1A). Our study aims to elucidate the role of *EDNRB* in HSCR susceptibility, explore the interplay between common and rare variants, and provide mechanistic insights into HSCR pathogenesis through functional studies.

**Figure 1:**
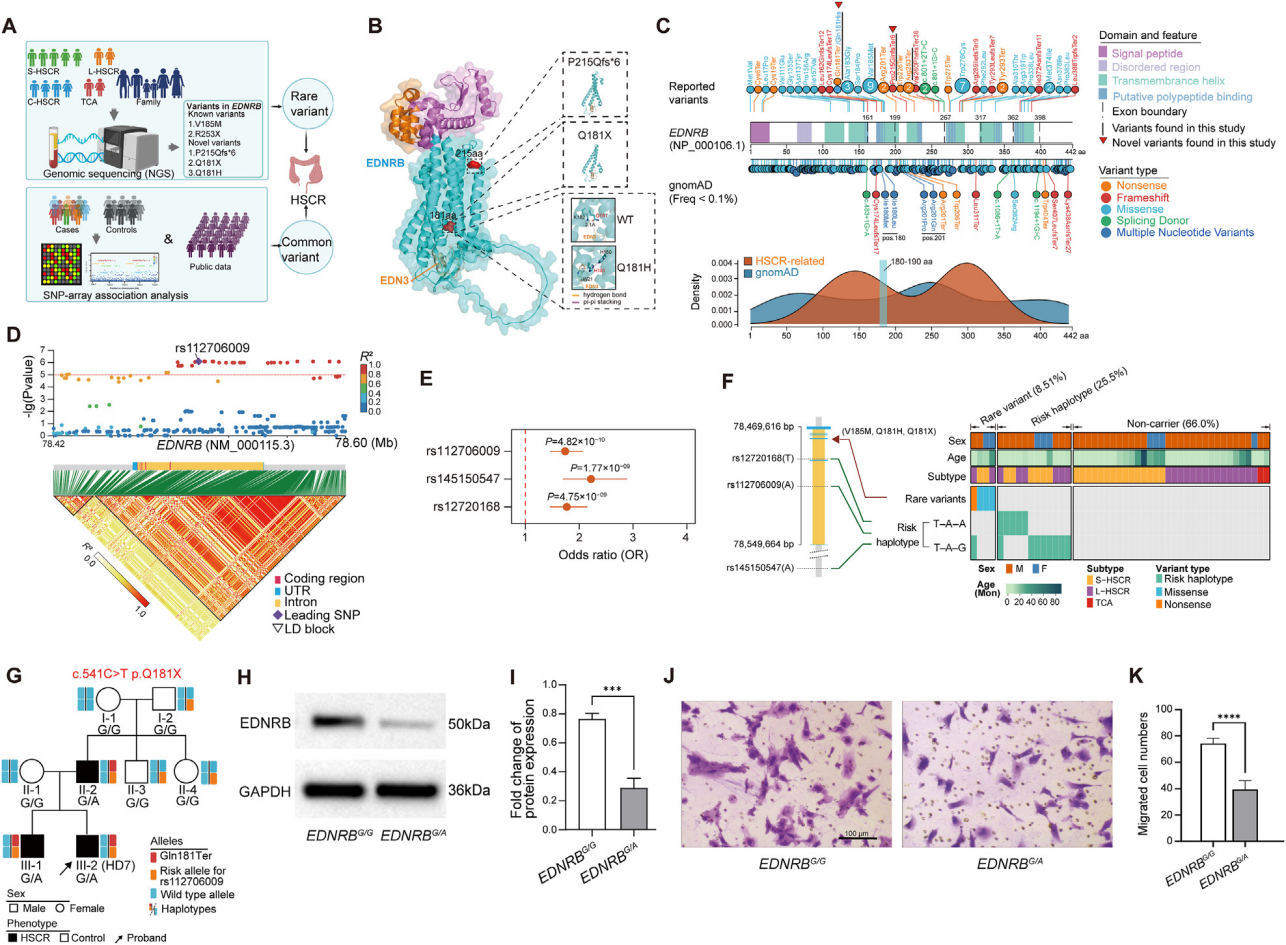
Comprehensive analysis of *EDNRB* rare and common variants in HSCR. **(A)** Overview of the genomic analysis for *EDNR*B variants in HSCR. **(B)** 3D structure shows the functional impact of three novel *EDNRB* variants. **(C)** The variant spectrum of *EDNRB* variants associated with HSCR. **(D)** Association results of common *EDNRB* variants. The upper panel is a regional association plot of *EDNRB* variants, with the leading single nucleotide polymorphism rs112706009 marked by a purple diamond. The lower panel shows the corresponding linkage disequilibrium (LD) structure (defined by pairwise R2) in the *EDNRB* genomic region. Darker colors indicate a stronger correlation between single nucleotide polymorphisms. **(E)** Association results for the three selected common variants incorporating additional controls. **(F)** Joint distribution of the common and rare EDNRB variants in 48 HSCR individuals who underwent genome sequencing. **(G)***EDNRB* p.Gln181Ter variant co-segregated with HSCR phenotype in a multi-generational family. **(H)** Western blot assay showed reduced EDNRB protein levels in heterozygous p.Gln181Ter ENCCs (*EDNRB*^G/A^). **(I)** Bar plots of gray value analysis of (H). **(J)** The migration ability of *EDNRB*^G/A^ ENCCs was evaluated using a transwell assay. Scale bar: 100 μm. **(K)** Bar plots of the number of migrated ENCCs for (J). Statistical significance is denoted by ∗∗∗∗ (*P* < 0.0001). EDNRB, endothelin receptor type B; HSCR, Hirschsprung disease; ENCC, enteric neural crest cells.

In this study, we recruited 535 HSCR patients and 2075 healthy controls from Guangzhou Women and Children's Medical Center. Of the patients, 372 (67.3%), 128 (23.1%), and 53 (9.6%) were diagnosed with short-segment HSCR, long-segment HSCR, and total colonic aganglionosis, respectively. The majority of HSCR cases were sporadic, with only one case (HD7) with familial aggregation. For genetic analysis, we selected 454 patients and all 2075 controls for SNP-array genotyping (Table S1). Additionally, 48 patients underwent genome sequencing (including HD7), and 51 patients underwent exome sequencing (Table S2).

The genome and exome sequencing of 100 HSCR participants revealed five rare coding variants in *EDNRB* [Gln181Ter, Gln181His, Val185Met (found in two participants), Pro215GlnfsTer6, and Arg253Ter], accounting for 6% of the cohort (Table S3). Three of these variants (Gln181Ter, Gln181His, and Pro215GlnfsTer6) were novel variants. Notably, we found two distinct variants at the 181th amino acid (181 aa) position (Gln181Ter and Gln181His), which were predicted to truncate protein or to alter EDNRB-EDN3 binding affinity, respectively (Fig. 1B). To contextualize these findings, we performed comprehensive curation and pathogenicity analysis on the spectrum of *EDNRB* variants associated with HSCR (Fig. 1C and Table S4). The HSCR-reported variants showed significantly higher pathogenicity scores (AM_pathogenicity, CADD_phred, and REVEL score) than background variants in the general population (Fig. S1), emphasizing their potential pathogenicity in HSCR. HSCR-reported variants, especially those protein-length-altering (nonsense, frameshift, and splicing), were concentrated in the third transmembrane helix domain. We further identified two vulnerable hotspots for HSCR-reported variants around 150 aa and 300 aa, both with high pathogenicity scores (Fig. 1D). Particularly, a notable region spanning 180–190 aa, which contains our identified recurrent variants at position 181 aa (Gln181Ter and Gln181His) and a previously reported recurrent variant (Val185Met), demonstrated high vulnerability to HSCR-related variations and exhibited strong concordance with pathogenicity scores.

To investigate whether common variants in *EDNRB* confer susceptibility to HSCR, we used SNP-array to obtain genotypes of 454 HSCR patients and 2075 controls. We also leveraged frequency data of additional controls (*n* = 12,959) from public databases to further assess the significance of candidate variants (supplementary methods). We observed genome-wide significant associations between common variants of *EDNRB* and HSCR susceptibility, with rs112706009 showing the strongest effect [odds ratio (95% confidence interval) = 1.74 (1.46–2.07), *P* = 4.82 × 10^−10^; Fig. 1D, E and Table S5]. We also identified two adjacent SNPs, rs145150547 and rs12720168, with weak to moderate linkage disequilibrium with rs112706009 (*r*^2^ = 0.27 and 0.71, respectively), showed significant associations (*P* = 1.77 × 10^−9^ and 4.75 × 10^−9^, respectively) (Fig. 1E and Table S5, 6). Epigenetic annotation revealed neuron-related histone markers surrounding the associated SNPs (Table S6), suggesting potential regulatory effects for these SNPs during enteric nervous system development. Haplotype analysis further confirmed the association of *EDNRB* common variants with HSCR risk, with rs12720168(T)-rs112706009(A)-rs145150547(G) and rs12720168(T)-rs112706009(A)-rs145150547(A) haplotypes showing significant HSCR association (Fig. S2). These findings demonstrate the role of common *EDNRB* variants in conferring HSCR susceptibility.

To address the interplays of common and rare variants, we performed haplotype phasing (rs12720168-rs112706009-rs145150547) on 48 HSCR participants who underwent genome sequencing. Our results revealed that the common and rare *EDNRB* variants contributed independently to HSCR risk (Fig. 1F). The joint consideration of both variant types substantially increased the proportion of EDNRB-explained HSCR cases from 6% to 34%. This dual contribution of common and rare variants to disease risk highlights the complex genetic architecture of HSCR and suggests that both variant types should be considered in the genetic risk assessment of HSCR.

Given that amino acid position 181 in *EDNRB* gene represents a mutational hotspot (p.Gln181Ter and p.Gln181His), we further investigated the functional implication of specific variants at this locus. We focused on the patient (HD7) with a heterozygous variant of p.Gln181Ter from a multiplex family of HSCR (Fig. 1G and Table S7). The proband (HD7), whose father (II-1) and brother (III-1) self-reported a HSCR history, presented with vomiting milk, yellow‒green gastric juice, and bloating 48 h after birth and was pathologically diagnosed with long-segment HSCR (Fig. S3A, B). Genome sequencing followed by Sanger sequencing confirmed a complete segregation of p.Gln181Ter and phenotype (Fig. S3C). This variant originated as a *de Novo* variant in participant II-3, predicted to truncate EDNRB protein at the conserved 181 aa (Fig. S4A, B). To investigate the functional impact of this variant on enteric neural crest cell (ENCC) development, we mimicked this heterozygous genotype in human induced pluripotent stem cells (denoted as *EDNRB*^G/A^) using gene-editing techniques and differentiated the mutated (*EDNRB*^G/A^) and wild-type (*EDNRB*^G/G^) human induced pluripotent stem cells into ENCCs (Fig. S4C). We found that p.Gln181Ter significantly reduced EDNRB expression at mRNA level (Fig. S4D) and protein level (Fig. 1H, I; Fig. S5). Functional assays revealed that this variant impaired the invasion velocity of ENCCs (Fig. 1J, K) and apparently reduced the migration ability of ENCCs (Fig. S4E–G). Thus, our functional assays prove the loss-of-function impact of p.Gln181Ter variant on HSCR pathogenesis.

In conclusion, our study comprehensively profiles the common and rare variants in *EDNRB*. Our findings of novel rare variants expand the understanding of the genetic landscape of HSCR. For the first time, we demonstrated a genome-wide significant association between common *EDNRB* variants and HSCR susceptibility. Importantly, we uncovered a synergistic effect between rare and common *EDNRB* variants, highlighting the importance of considering both common and rare variants in understanding HSCR etiology. Thus, we argue that the role of *EDNRB* in HSCR risk may have been significantly underestimated, warranting further investigations considering both rare and common variants in genetic risk assessment. This approach may serve as a model for investigating other complex genetic disorders, potentially leading to more personalized diagnostic and therapeutic strategies.

## CRediT authorship contribution statement

**Xiaoyu Zuo:** Writing – review & editing, Writing – original draft, Visualization, Project administration, Methodology, Investigation, Formal analysis, Data curation. **Jinglu Zhao:** Writing – original draft, Validation, Investigation, Funding acquisition, Data curation. **Zipeng Liu:** Writing – original draft, Software, Methodology, Formal analysis. **Yuxiao Yao:** Validation, Investigation. **Dingyang Li:** Visualization, Resources. **Xiaogang Xu:** Investigation, Data curation. **Huimin Xia:** Supervision, Funding acquisition. **Lihua Huang:** Validation, Resources, Funding acquisition. **Jixiao Zeng:** Supervision, Funding acquisition. **Yan Zhang:** Writing – review & editing, Supervision, Funding acquisition, Conceptualization.

## Ethics declaration

Written informed consents were obtained from all participants. All procedures performed in studies involving human participants were in accordance with the ethical standards of the institutional review board of Guangzhou Women and Children's Medical Center (No. 13701) and with the 1964 Helsinki Declaration and its later amendments or comparable ethical standards.

## Data availability

Individual-level genetic data can be made available upon reasonable request. All data sharing will be conducted in strict accordance with the Regulations on the Management of Human Genetic Resources in China and other applicable laws and regulations. Researchers who are interested in collaboration are warmly welcome to contact the corresponding author (yannizy@gwcmc.org (Z.Y.)).

## Funding

This study was founded by the 10.13039/501100001809National Natural Science Foundation of China (No. 81970450 to Z.Y.; 82201893 to Z.J.L.; 82070528 to H.L.H.; 82170528 to Z.J.X.), the 10.13039/501100003453Natural Science Foundation of Guangdong Province, China (No. 2022A1515012254, 2018A030313570 to Z.J.X.), the 10.13039/501100021171Guangdong Basic and Applied Basic Research Foundation (No. 2021A1515220146 to Z.Y.), the Guangzhou Basic Research Plan City School (Institute) Enterprise Joint Funding Project (China) (No. SL2024A03J01 to Z.Y.), the 10.13039/501100012245Science and Technology Planning Project of Guangdong Province, China (No. 2019B020227001 to X.H.M.), and the Science and Technology Project of Guangzhou, China (No. 202206080002 to X.H.M.).

## Conflict of interests

The authors declared no competing interests.
