# Supplementary material for: Integrated genomic analysis of EDNRB common and rare variants in Hirschsprung disease
Source: Genes Dis. 2025 Mar 4;13(2):101595. doi: 10.1016/j.gendis.2025.101595 (PMC12596629; doi:10.1016/j.gendis.2025.101595)
Supplement: Multimedia component 1 [file mmc1.pdf]

Supplementary material and methods

Participants

We recruited a large cohort of Hirschsprung disease (HSCR) patients comprising 535 participants from Guangzhou Women and Children's Medical Center between 2017 to 2020. Inclusion criteria for HSCR patients were as follows: (1) histologically confirmed HSCR by biopsy specimens with the absence of the enteric ganglia; (2) age <18 years; (3) claimed to be southern Chinese; (4) isolated HSCR with no syndromic manifestation. Patients with syndromic HSCR were excluded. We also recruited 2,075 healthy controls from physical examination center at the same medical center. The majority of our HSCR cases were sporadic, with only one participant has multiple affected members across generations. This study was approved by the Ethics Committee of Guangzhou Women and Children's Medical Center. Written informed consent was obtained from all participants or their legal guardians.

For genetic analysis, we selected 454 HSCR patients and all 2,075 controls for SNP-array genotyping (**Table S1**). Additionally, 48 patients underwent genome sequencing (including the proband from the multiplex family, III-1), and 51 patients had exome sequencing performed (**Table S2**).

In the case of the multiplex family (**Table S7**), genome sequencing was extended to the family members. The proband in this family was diagnosed with long-segment HSCR (L-HSCR) through barium enema and histological examination, while the proband's father (II-2) and brother (III-1) self-reported prior HSCR diagnosis histories.

Exome- and genome- sequencing

Participants' peripheral blood samples were extracted using the Blood Genomic DNA Extraction Kit (TIANGEN, Cat. No. DP348-03). Exome sequencing (WES) library was constructed using Agilent SureSelect v6 capture kits, then was paired-end sequenced of 150bp on a Illumina HiSeq 2500 system (Illumina, Inc., San Diego,

USA). Genome sequencing (WGS) was conducted on a DNBSEQ-T7 sequencing platform (MGI Tech Co., Ltd., Shenzhen, China) to generate paired-end 150bp reads.

Sequence reads were quality controlled by fastp (0.23.4) and mapped to the GRCh37/hg19 reference genome using bwa (v.0.7.12). Genome Analysis Toolkit (version 4.1.3)[1] was used for subsequent variant calling according to the Best Practices recommendations. ANNOVAR[2] was then applied for variant annotation. If three or more annotations from SIFT[3], PROVEAN[4], MutationTaster[5], FATHMM[6], CADD[7] and Polyphen2[8] were predicted to be deleterious, the variants were considered pathogenic.

Independent cohorts of southern Chinese population

There publicly available large-scale databases consisting of the independent cohorts of southern Chinese population were incorporated in this study, including GDBIG (Genome Database of Born In Guangzhou Cohort study, http://gdbig.bigcs.com.cn), WBBC (Westlake BioBank for Chinese, https://wbbc.westlake.edu.cn/) and PGG.Han2.0, https://www.biosino.org/pgghan2/index). 1,665 unrelated samples in GDBIG, 4,044 samples of south China, and 7,250 South coast Han samples in PGG.Han were included, resulting in a combined cohort of 12,959 southern Chinese individuals. The minor allele count (MAC) and minor allele frequency (MAF) reported by these databases were retrieved and combined.

Association analysis of *EDNRB* common variants

The genotyping process was executed utilizing Infinium Asian Screening Array-24 v1.0 BeadChip arrays (Illumina, Inc., San Diego, CA, USA) in accordance with the guidelines provided by the manufacturer. Quality control was carried out employing PLINK (v1.9b, www.cog-genomics.org/plink/1.9/)[9]. The parameters for low sample quality encompassed a genotype call rate of less than 92%, issues with sex estimation, a high heterozygosity rate (exceeding 3 standard deviations), and hidden relatedness. Samples deemed to be of low quality were eliminated from the study. Population stratification was assessed through principal component analysis (PCA). We excluded

low-quality SNPs that had genotyping rates below 95%, while also filtering out those that did not meet Hardy-Weinberg equilibrium (HWE, P value < 1×10^{-4}). SNP imputation was conducted using IMPUTE2[10], with 1000 Genomes Phase 3 (October 2014, NCBI build b37) serving as the reference panel. Post-imputation, only SNPs with an INFO score greater than 0.85 and an accuracy score exceeding 0.9 were retained. The association analysis was performed via SNPTEST [11] (v2.5.6, https://www.well.ox.ac.uk/~gav/snptest) utilizing the parameters "-method score -frequentist 1". Sex and the first three principal components were included as covariates. The association results were illustrated through a regional association plot and LD block using either the Locuszoom [12] or LDBlockShow [13] program. By merging public data, the consistency of the minor allele frequency (MAF) in controls from the current cohort was evaluated against that in the combined controls from three public datasets via the $\chi2$ test. Additionally, the $\chi2$ test was applied for the allelic association assessment of the top *EDNRB* signals by incorporating the combined samples from our cohort along with the public datasets.

Haplotype estimation and association analysis

Haplotypes spanning the top three significant SNPs with LD R^2 < 0.8 (rs12720168, rs112706009, rs145150547), and their frequencies were estimated. Shapeit software (v2.r904) was used for haplotype phasing of all SNPs within 50kb flanking of *EDNRB* gene. Only haplotypes with frequency higher than 1% in either cases or controls were included. The association of these haplotypes with the trait was examined using $\chi2$ test (chisq.test() function in R) by treating C-T-G haplotype as reference. Odds ratios (OR) and P-values were estimated to determine the statistical significance. The impact of rare variants and risk haplotypes on the trait was evaluated. In this analysis, only 48 participants who underwent genome-sequencing were included due to the completeness of the both the rare and common variants data in these individuals. In these individuals, rare variants p.Gln181His, p.Gln181Ter and p.Val185Met were identified. A heatmap visualization was created to compare the distribution of variants

among carriers and non-carriers, including sex, age, and subtype information. Data analysis and visualization were performed in R software (v4.1.3).

Curation of the spectrum of mutations in EDNRB

To build the spectrum of mutations in *EDNRB*, we curated data from both public databases and publications. The databases included ClinVar (www.ncbi.nlm.nih.gov/clinvar/), OMIM (www.omim.org) and gnomAD (www.gnomad-sg.org/). For ClinVar and OMIM, we collected likely pathogenic and pathogenic mutations reported in *EDNRB*-related diseases such as HSCR, Waardenburg syndrome, and ABCD syndrome. We curated rare variants in *EDNRB* (NM_000115) with minor allele frequency < 0.001 in the gnomAD database as a background spectrum. Missense, nonsense, splicing donor, and frameshift variants were included. Missense variants affecting the same amino acid were labeled "MNV". Furthermore, to collect reported mutations from publications, a literature search for Hirschsprung, Waardenburg syndrome and ABCD syndrome was conducted using PubMed (https://pubmed.ncbi.nlm.nih.gov/) and Google Scholar (https://scholar.google.com/) of articles published on HSCR between 1980 and 2022 and their references. Two researchers independently curated the variants and reannotated them using the transcript NM_000115. The spectrum plot was generated by ProteinPaint (proteinpaint.stjude.org/) by using NP_000106.1 as the EDNRB model. Information on protein domains was retrieved from InterPro (www.ebi.ac.uk/interpro/protein/UniProt/P24530/).

Sanger sequencing

Primers for human *EDNRB* DNA sequencing were used at 10 μM. The forward sequence is AAGCAAGCAGATTCGCAGAT, and the reverse sequence is GGAAGTCTGGTCTCCTCAGC. PCR amplification was carried out using 2 × Rapid Taq Master Mix (Vazyme, Cat No. P222-02) and under the conditions recommended by the manufacturer. The PCR products were resequenced by PCR for target site detection at Beijing Liuhe Huada Gene Technology Co., Ltd.

Protein Structure Analysis of *EDNRB* Variants

To analyze the spatial distribution and functional impact of *EDNRB* variants, we employed computational tools. Spatial distribution was assessed with R and ggplot2. Tertiary protein structure data from the PDB (PDB: 6igk) was visualized and classified using PyMOL (Version 2.5.4) installed via Conda. Data sources included a complete amino acid sequence for EDNRB (NP_000106.1). Variants were categorized based on their effects on protein interactions, including hydrogen bonds and π-π stacking. Hydrogen bond analysis highlighted potential changes in binding affinity and structural stability. π-π stacking interactions were investigated for their impact on protein stability and function. The analysis involved mapping variants onto the 3D structure of EDNRB, generating visualizations to depict their positions and interactions. Key variants such as p.Pro215GlnfsTer6, p.Gln181Ter and p.Gln181His were annotated for their significant structural and functional implications.

Cell culture and *EDNRB* point mutation strategy

Human induced pluripotent stem cell (hiPSC) and enteric neural crest cell (ENCC) differentiation was performed as previously described[14]. Briefly, hiPS cells were cultured on Matrigel-coated plates (Corning, Cat No. 35354234) in mTeSR1 medium (Stemcell, Cat No. 85851) and then were replaced with ENCC differentiation medium (neurobasal medium, DMEM-F12, N2, B27 containing L-GLU, β-ME, FGF2, EGF) at 37 °C in 5% CO_2. The cells were passaged as clumps every 3-4 days using Accutase (Sigma, Cat No. A6964). We generated heterozygous *EDNRB* p.Q181X lines ($EDNRB^{G/A}$ hiPSCs) by using cytosine base editors (CBEs) with a mutation-specific sgRNA (TTCATACAGAAAGCCTCCGT). $EDNRB^{G/A}$ hiPSCs were subsequently induced into ENCCs by replacing the maintenance medium with differentiation medium. $EDNRB^{G/A}$ ENCCs were used for further functional assays.

Immunocytochemistry, RNA isolation, qRT–PCR and Western blot of ENCC

Cells were fixed with 4% paraformaldehyde/PBS at 4 °C for 15 minutes, washed twice with PBS, incubated with 0.3% Triton X-100 for 30 minutes, and blocked with 5% BSA/PBS for 1 hour. Nuclei were counterstained with DAPI. Images were acquired with a Leica DM4-B fluorescence microscope (Leica, Germany). The antibodies used are listed in **Supplementary resources**. RNA isolation, qRT-PCR (quantitative reverse-transcription polymerase chain reactionquantitative real-time, cell lysis, protein quantification, and Western blot were performed as previously described[15]. The primary and secondary antibodies used are described in **Supplementary resources**.

Cell migration and invasion

ENCC migration was measured by wound-healing assay. Briefly, ENCCs were plated at 5 × 10^5 cells per ml in each Matrigel-coated 12-well cell culture plate (Corning, Costar 3513), grown to full confluency and growth arrested. Following a 24 h growth-arresting period, the cell wound was scratched using the Scratcher tool (SPL LIFESCIENCES, Cat No. 201925), 1 ml of fresh NB+ medium was added to the culture dish, and incubation continued for 48 h. The migrated cells were captured with a Leica DMi-8 inverted epifluorescence microscope (Leica, Germany) at 0 h, 24 h, and 48 h.

ENCC invasion was measured using a Transwell chamber method. ENCCs were seeded in NB+ medium and plated on Matrigel-coated 8 μm cell culture inserts (Corning, Cat No. 353097) at 2 × 104 cells per insert. The cells were incubated at 37°C for 48 hours in pure NB+ medium. After lifting the inserts, the upper surface of the membrane was cleaned with a cotton swab, and the membrane was fixed in methanol and stained with crystal violet. (Tianjin Damao, Cat No. 548-62-9). The crystal violet-positive cells on the lower surface of the membrane were counted under an inverted Leica DMi-8 inverted epifluorescence microscope (Leica, Germany), and cell migration was expressed as the number of cells per field of view.

Statistical analysis

To determine the number and migration distance of enteric nerve cells present, a 10-segment length of the gut to the vent was counted. The cell numbers and migration distance are presented as the mean ± SEM for at least 10 separate zebrafish per group. Images were acquired with a Leica SP8 confocal microscope (Leica, Germany). All results were recorded and analyzed using GraphPad Prism 9.0. Student's t test (2-tailed) was mainly used as the statistical analysis method. Statistical significance was defined as a P value < 0.05 .

Reference

1. DePristo MA, Banks E, Poplin R, et al. A framework for variation discovery and genotyping using next-generation DNA sequencing data. Nat Genet. 2011;43(5):491-498.
2. Wang K, Li M, Hakonarson H. ANNOVAR: functional annotation of genetic variants from high-throughput sequencing data. Nucleic Acids Res. 2010;38(16):e164.
3. Ng PC, Henikoff S. SIFT: Predicting amino acid changes that affect protein function. Nucleic Acids Res. 2003;31(13):3812-3814.
4. Choi Y, Sims GE, Murphy S, Miller JR, Chan AP. Predicting the functional effect of amino acid substitutions and indels. PLoS One. 2012;7(10):e46688.
5. Schwarz JM, Cooper DN, Schuelke M, Seelow D. MutationTaster2: mutation prediction for the deep-sequencing age. Nat Methods. 2014;11(4):361-362.
6. Shihab HA, Gough J, Cooper DN, et al. Predicting the functional, molecular, and phenotypic consequences of amino acid substitutions using hidden Markov models. Human mutation. 2013;34(1):57-65.
7. Rentzsch P, Schubach M, Shendure J, Kircher MJGM. CADD-Splice—improving genome-wide variant effect prediction using deep learning-derived splice scores. 2021;13(1).
8. Adzhubei IA, Schmidt S, Peshkin L, et al. A method and server for predicting damaging missense mutations. Nat Methods. 2010;7(4):248-249.
9. Chang CC, Chow CC, Tellier LC, Vattikuti S, Purcell SM, Lee JJ. Second-generation PLINK: rising to the challenge of larger and richer datasets. Gigascience. 2015;4:7.
10. Howie BN, Donnelly P, Marchini J. A flexible and accurate genotype imputation method for the next generation of genome-wide association studies. PLoS Genet. 2009;5(6):e1000529.
11. Marchini J, Howie B. Genotype imputation for genome-wide association studies. Nature reviews Genetics. 2010;11(7):499-511.
12. Pruim RJ, Welch RP, Sanna S, et al. LocusZoom: regional visualization of genome-wide association scan results. Bioinformatics. 2010;26(18):2336-2337.
13. Dong SS, He WM, Ji JJ, Zhang C, Guo Y, Yang TL. LDBlockShow: a fast and convenient tool for visualizing linkage disequilibrium and haplotype blocks based on variant call format files. Brief Bioinform. 2021;22(4).

14. Li W, Huang L, Zeng J, et al. Characterization and transplantation of enteric neural crest cells from human induced pluripotent stem cells. Molecular psychiatry. 2018;23(3):499-508.
15. Zhao JL, Zhao YY, Zhu WJ. A high-fat, high-protein diet attenuates the negative impact of casein-induced chronic inflammation on testicular steroidogenesis and sperm parameters in adult mice. General and comparative endocrinology. 2017;252:48-59.

Supplementary resources

REAGENT OR RESOURCE	SOURCE	IDENTIFIER
**1. Antibodies**		
HuC/HuD Monoclonal Antibody (16A11)	ThermoFisher	A21271
Alexa Fluor 568 goat anti mouse IgG	Abcam	ab175473
Anti-GAPDH antibody [6C5] – Loading Control	Abcam	ab8245
Goat Anti-Mouse IgG H&L (HRP)	Abcam	ab205719
Anti-Endothelin B Receptor/ET-B antibody	Abcam	ab129102
Goat Anti-Rabbit IgG H&L (Alexa Fluor® 488)	Abcam	ab150077
Goat Anti-Rabbit IgG H&L (HRP)	Abcam	ab205718
**2. Other reagents and consumables**		
Blood Genomic DNA Extraction Kit	TIANGEN	DP348-03
2 × Rapid Taq Master Mix	Vazyme	P222-02
TritonX-100	Sigma-Aldrich	T8787
DMSO	MPBIO	196055
VECTASHIELD with DAPI	Vectorlabs	H-1200
BES-H2O2-Ac solution	Wako	028-17811
1-phenyl-2-thiourea	Sigma-Aldrich	P7629
tricaine methanesulfonate,	Sigma-Aldrich	E10521
methylcellulose	Sigma-Aldrich	217277
Matrigel-coated plates	Corning	354234
mTeSR1 medium	Stemcell	85851
Neurobasal medium	Cellapy	CA1001500
DME/F-12	Cytiva	SH30023.01
nerve growth factor (N2)	Stemcell	07152
B27	Gibco	17504-044
L-GLU	Sigma-Aldrich	G8540
2ME	Gibco	21985-023
b-FGF	Stemcell	78003
EGF	Stemcell	78006.1
Accutase	Sigma-Aldrich	A6964
BSA	FUDE biological	FD0030-10
Matrigel-coated 12-well cell culture plate	Corning	Costar 3513
Scrather tool	SPL LIFESCIENCE	201925
Matrigel-coated 8μm cell culture inserts	Corning	353097
Crystal violet	Tianjin Damao	548-62-9
Pageruler prestained protein ladder (10-180kDa)	Thermo Scientific	26616
Prestained protein marker II (10-200 kDa)	Servicebio	G2058
**3. Cell model: Organisms/strains**		

Human iPSCs	Center for Stem Cell Biology and Tissue Engineering, Key Laboratory for Stem Cells and Tissue Engineering, Ministry of Education, Sun Yat-Sen University	N/A
**4. Oligonucleotides**		
Amplification primers for *EDNRB* (Fwd-AAGCAAGCAGATTCGCAGAT, Rev- GGAAGTCTGGTCTCCTCAGC)	IDT	N/A
Sanger sequencing primers for *EDNRB* (Fwd- GGCATACCTTAGTTTTATTG , Rev- GGCATCTTATGAAACCC)	IDT	N/A
**5. Software and algorithms**		
Genome Analysis Toolkit	version 3.4	https://gatk.broadinstitute.org
PLINK	v1.9b	www.cog-genomics.org/plink/1.9/
Chromas	Chromas Lite	chromas.updatestar.com/
GraphPad Prism software version 10.1.2	GraphPad Software, Inc.	https://www.graphpad.com/

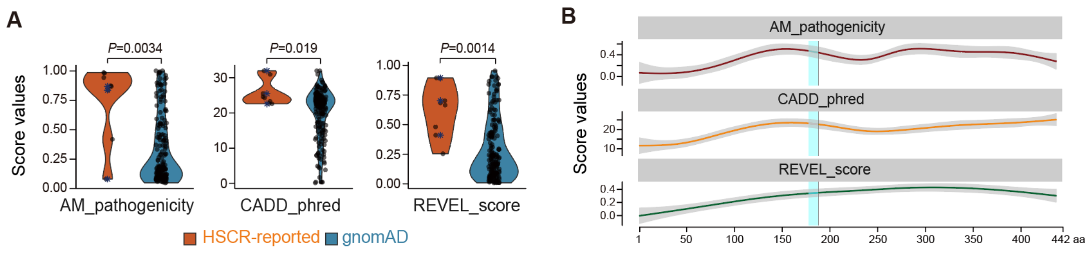

Fig. S1. Pathogenicity score analysis of HSCR-reported *EDNRB* variants.
(A) Comparison of the pathogenicity scores (AM_pathogenicity, CADD_phred, and REVEL score) between HSCR-reported and gnomAD variants; **(B)** AM_pathogenicity, CADD_phred, and REVEL score for all the *EDNRB* variants.

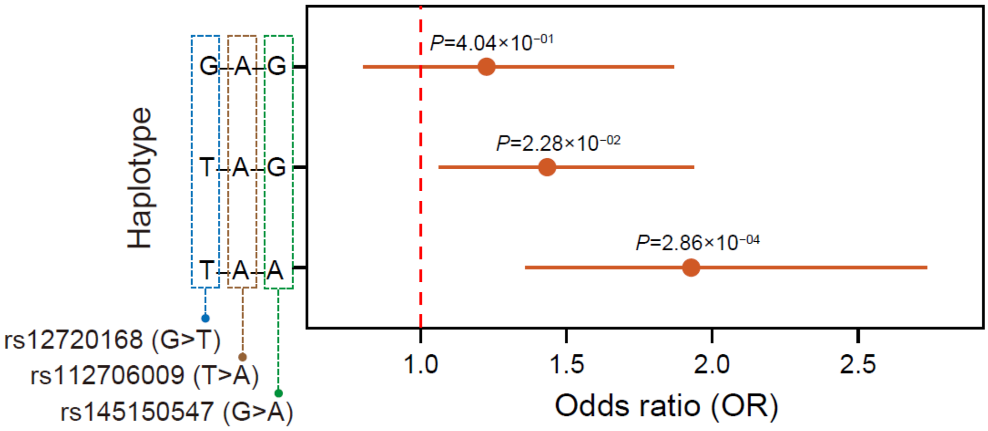

Fig. S2. Haplotype association analysis for the common SNPs with HSCR.

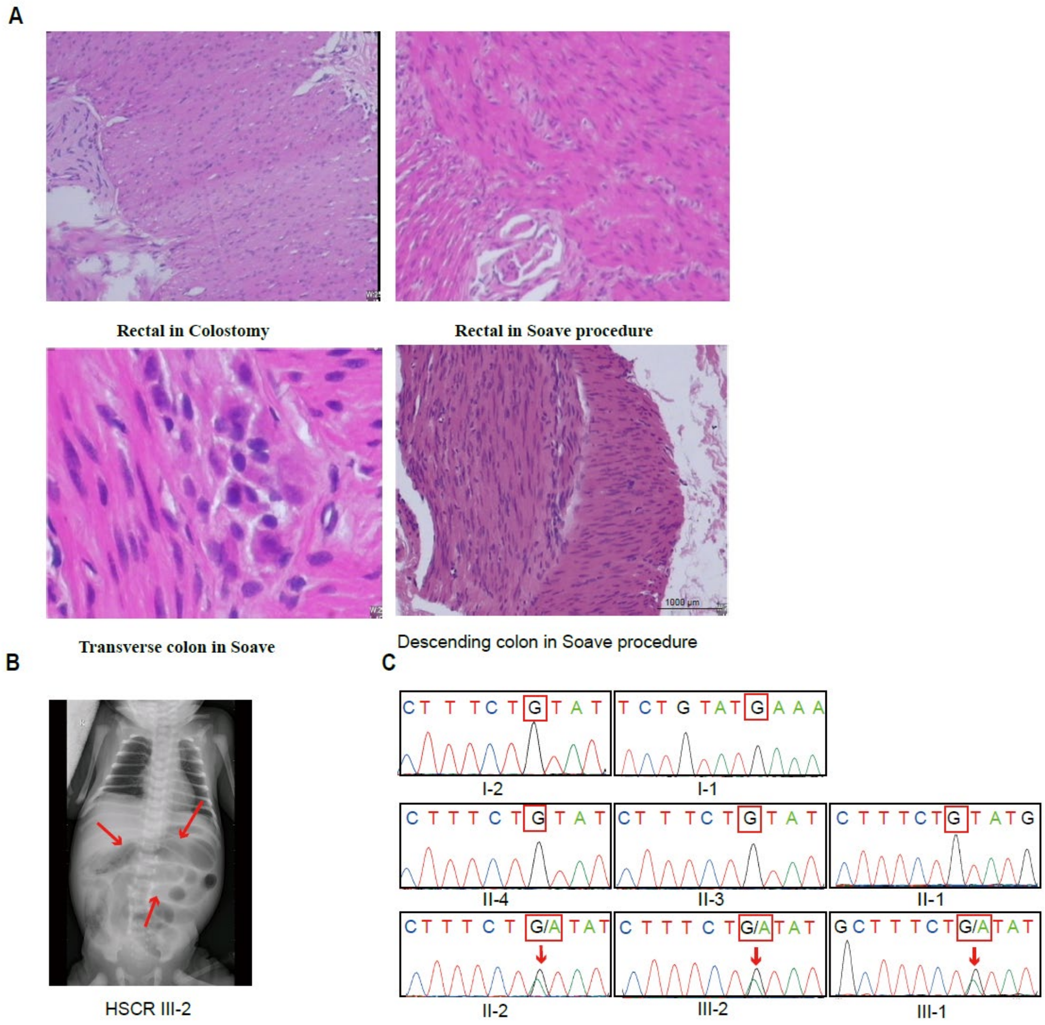

Fig. S3. A truncated mutation p.Gln181Ter in EDNRB was identified from the HSCR pedigree.

(A) Pathological staining images of the proband's colon. Scale bar: 1000 μm; **(B)** Abdominal radiograph of the proband (III-1) in the family; **(C)** Sanger sequencing confirmed the p.Q181X mutation.

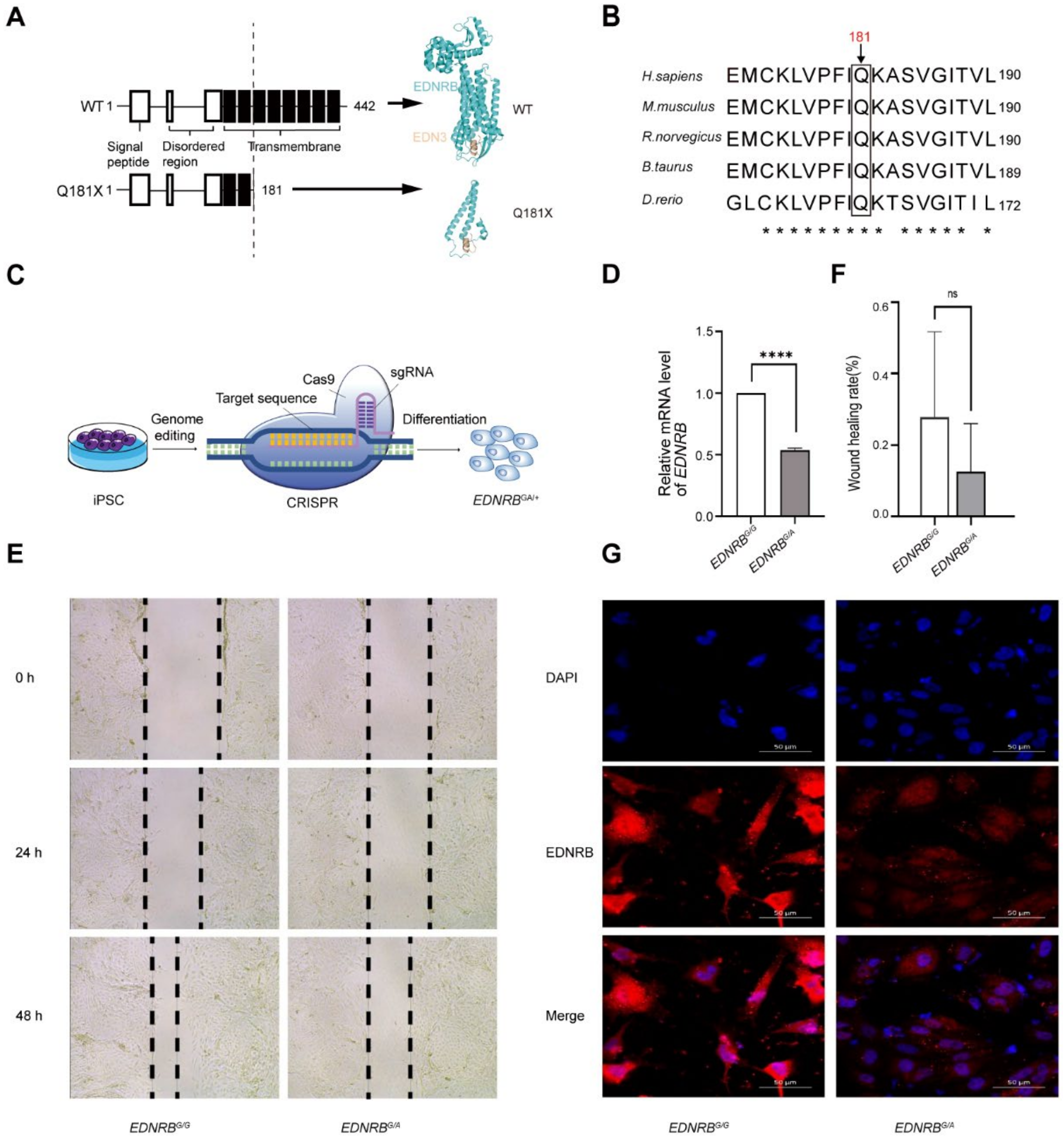

Fig. S4. Functional assays of EDNRB p.Gln181Ter variant.

(A) The diagram depicts the effect of the p.Gln181Ter variant to produce truncated EDNRB protein; (**B**) The p.Gln181Ter variant is conserved across species; **(C)** Schematic diagram of gene editing at the *EDNRB* locus to obtain a heterozygous p.Gln181Ter variant in hiPSCs and differentiation from hiPSCs to ENCCs (*EDNRB*$^{G/A}$); **(D)** *EDNRB* mRNA expression was decreased in *EDNRB*$^{G/A}$ ENCCs; **(E)** Wound healing assay was done to evaluate the migration potential of *EDNRB*$^{G/A}$ ENCCs. Cells were wounded and monitored with a microscope every 24 hours; **(F)** *EDNRB*$^{G/A}$ ENCCs showed slower healing rate; **(G)** Immunofluorescent localization of EDNRB proteins in the *EDNRB*$^{G/A}$ ENCC.

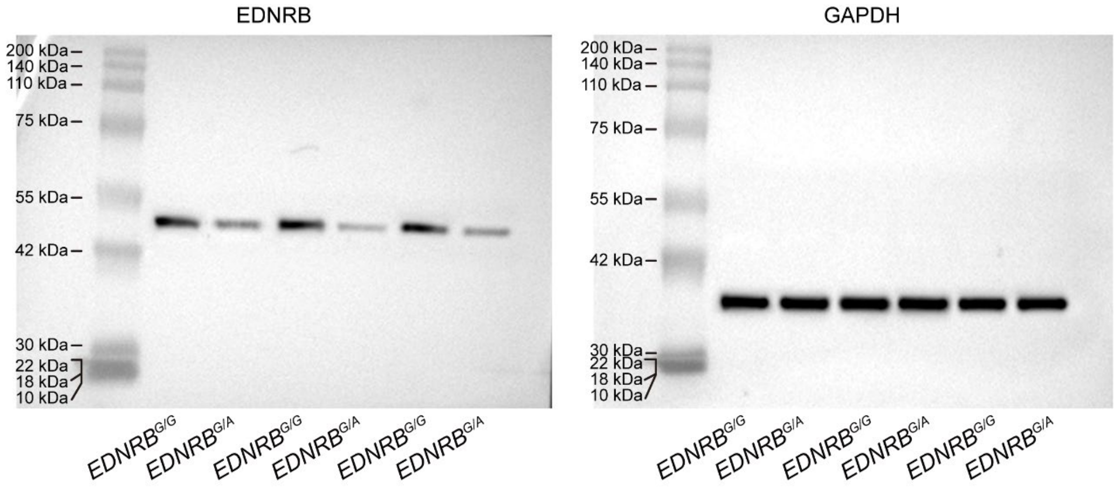

Fig. S5. Full western blotting images for Fig. 1H

Table S1. Characteristics of participants for association study.

	Cases	Controls	*P*
n	454	2075	
Gender			<0.0001
Females	82(18.06%)	666(32.10%)	
Males	372(81.94%)	1409(67.90%)	
Subtype			
S-HCSR	317 (69.82%)	N/A	
L-HCSR	92 (20.26%)	N/A	
TCA	45 (9.91%)	N/A	

Note: S-HSCR: short-segment HSCR; L-HSCR: long-segment HSCR; TCA: Total colonic aganglionosis; N/A denotes that the variable is not suitable for controls. *p* values for association between the cases group and control group.

Table S2. Summary of clinical information in exome and genome sequencing cohort.

	Exome-sequencing participants	Genome-sequencing participants
n	51	48
Age (Month)	12.71±19.9	13.5±15.04
Gender		
Females	9	9
Males	42	39
Subtype		
S-HCSR	31	24
L-HCSR	14	22
TCA	6	2

Note: Age: Mean±SD; S-HSCR: short-segment HSCR; L-HSCR: long-segment HSCR; TCA: total colonic aganglionosis;

Table S3. Rare *EDNRB* variants identified in children of Hirschsprung disease by exome sequencing and genome sequencing

Individual	Sequencing	Sex	Age (m)	Subtype	Sporadic	Chr	Position (bp) [a]	Ref/Alt	Function	Protein change	dbSNP [b]	PAF [c]	reported [d]	Pathogenicity [e]
HD1	WGS	F	12	S-HSCR	Yes	13	78477673	C/T	Missense	p.Val185Met	rs781214034	1.31E-03	Yes	Uncertain significance
HD2	WGS	M	14	S-HSCR	Yes	13	78477673	C/T	Missense	p.Val185Met	rs781214034	1.31E-03	Yes	Uncertain significance
HD3	WES	F	12	L-HSCR	Yes	13	78477673	C/T	Missense	p.Val185Met	rs781214034	1.31E-03	Yes	Uncertain significance
HD4	WES	M		S-HSCR	Yes	13	78477335	G/A	Nonsense	p.Arg253Ter	rs104894390	n.a	Yes	Pathogenic
HD5	WGS	F	36	L-HSCR	Yes	13	78477683	C/A	Missense	p.Gln181His	N/A	n.a	No	Uncertain significance
HD6	WES	M	23	L-HSCR	Yes	13	78477447	TG/T	Frameshift	p.Pro215GlnfsTer6	N/A	n.a	No	Pathogenic
HD7 (III-1)	WGS	M	6	L-HSCR	No	13	78477683	C/A	Nonsense	p.Gln181Ter	N/A	n.a	No	Pathogenic

[a] position is based on UCSC hg19 genome; [b] dbSNP (v150) identifier; [c] population allele frequency in eastern Asian population (gnomAD v2.1.1); [d] previously reported in Clinvar, literature, or population databases (gnomAD or 1KGP, etc.); [e] pathogenicity is assessed under ACMG guideline. Abbrevations: WES: exome sequencing; WGS: genome sequencing; F: Female, M: Male; S-HSCR: short-segment HSCR; L-HSCR: long-segment HSCR; Ref: reference allele; Alt: alternate allele; n.a: not available in database.

Table S4. Full list of the curated *EDNRB* variants used in spectrum analysis.

Mutation	Pos	Classification	Source	Mutation	Pos	Classification	Source	Mutation	Pos	Classification	Source
**Cys6Ter**	6	Nonsence	Clinvar	**Thr77Arg**	77	mnv	gnomAD	**Ser250Ile**	250	mnv	gnomAD
**Cys19Ter**	19	Nonsence	Clinvar	**Thr77Met**	77	mnv	gnomAD	**Leu252Pro**	252	Missence	gnomAD
**Cys174LeufsTer17**	174	Frameshift	Clinvar	**Gly79Ala**	79	Missence	gnomAD	**Arg253Gln**	253	Missence	gnomAD
**Ala183Gly**	183	Missence	Clinvar	**Ser80Phe**	80	Missence	gnomAD	**Ile254Asn**	254	mnv	gnomAD
**Ser184Pro**	184	Missence	Clinvar	**Pro81Leu**	81	Missence	gnomAD	**Ile254Leu**	254	mnv	gnomAD
**Val185Met**	185	Missence	Clinvar	**Arg83Cys**	83	Missence	gnomAD	**Ile254Thr**	254	mnv	gnomAD
**Arg201Ter**	201	Nonsence	Clinvar	**Thr84Asn**	84	mnv	gnomAD	**His258Asp**	258	mnv	gnomAD
**Arg253Ter**	253	Nonsence	Clinvar	**Thr84Ile**	84	mnv	gnomAD	**His258Gln**	258	mnv	gnomAD
**Val260PhefsTer36**	260	Frameshift	Clinvar	**Pro87His**	87	mnv	gnomAD	**His258Tyr**	258	mnv	gnomAD
**Trp276Cys**	276	Missence	Clinvar	**Pro87Ser**	87	mnv	gnomAD	**Pro259Arg**	259	mnv	gnomAD
**Pro338Leu**	338	Missence	Clinvar	**Pro88Leu**	88	Missence	gnomAD	**Pro259Ser**	259	mnv	gnomAD
**c.801+2T>C**	267	splicing	Clinvar	**Pro89Arg**	89	Missence	gnomAD	**Val260Gly**	260	mnv	gnomAD
**c.801+2T>C**	267	splicing	Clinvar	**Cys90Phe**	90	Missence	gnomAD	**Val260Ile**	260	mnv	gnomAD
**Val185Met**	185	Missence	Clinvar	**Gly92Glu**	92	Missence	gnomAD	**Val260Phe**	260	mnv	gnomAD
**Val185Met**	185	Missence	Clinvar	**Ile94Val**	94	Missence	gnomAD	**Ala264Val**	264	Missence	gnomAD
**Arg201Ter**	201	Nonsence	Clinvar	**Glu95Val**	95	Missence	gnomAD	**Phe268Leu**	268	Missence	gnomAD
**Trp276Cys**	276	Missence	Clinvar	**Lys97Glu**	97	Missence	gnomAD	**Lys270Gln**	270	Missence	gnomAD
**Trp275Ter**	275	Nonsence	Clinvar	**Thr99Pro**	99	Missence	gnomAD	**Asp274Asn**	274	mnv	gnomAD
**Tyr293LeufsTer7**	293	Frameshift	Clinvar	**Asn104Ile**	104	Missence	gnomAD	**Asp274Gly**	274	mnv	gnomAD
**Val185Met**	185	Missence	Clinvar	**Thr105Met**	105	Missence	gnomAD	**Asp274Tyr**	274	mnv	gnomAD
**Trp276Cys**	276	Missence	Clinvar	**Val111Glu**	111	Missence	gnomAD	**Asp274Val**	274	mnv	gnomAD
**Trp276Cys**	276	Missence	Clinvar	**Val113Leu**	113	mnv	gnomAD	**Trp275Ser**	275	Missence	gnomAD

**Met1Val**	1	Missence	PMID: 23840513	**Val113Met**	113	mnv	gnomAD	**Ser279Asn**	279	mnv	gnomAD
**Leu17Pro**	17	Missence	PMID: 28236341	**Ile116Thr**	116	Missence	gnomAD	**Ser279Thr**	279	mnv	gnomAD
**Val111Glu**	111	Missence	PMID: 17009072	**Arg124Lys**	124	Missence	gnomAD	**Phe291Leu**	291	Missence	gnomAD
**Asn137Tyr**	137	Missence	PMID: 28236341	**Tyr127Phe**	127	Missence	gnomAD	**Tyr293His**	293	Missence	gnomAD
**Pro156Arg**	156	Missence	PMID: 28236341	**Lys130Thr**	130	Missence	gnomAD	**Thr294Ile**	294	Missence	gnomAD
**Ile157Val**	157	Missence	PMID: 31544051	**Cys131Arg**	131	Missence	gnomAD	**Leu301Ser**	301	Missence	gnomAD
**Leu162GlnfsTer12**	162	Frameshift	PMID: 28236341	**Gly135Ser**	135	Missence	gnomAD	**Lys303Arg**	303	Missence	gnomAD
**Gln181Ter**	181	Nonsence	this study	**Ile138Val**	138	Missence	gnomAD	**Lys304Asn**	304	mnv	gnomAD
**Ala183Gly**	183	Missence	PMID: 8634719	**Leu139Phe**	139	mnv	gnomAD	**Lys304Ile**	304	mnv	gnomAD
**Ala183Gly**	183	Missence	PMID: 8634719	**Leu139Val**	139	mnv	gnomAD	**Gln308Lys**	308	Missence	gnomAD
**Val185Met**	185	Missence	PMID: 29106856	**Ala141Ser**	141	mnv	gnomAD	**Leu311Ter**	311	Frameshift	gnomAD
**Val185Met**	185	Missence	PMID: 17554617	**Ala141Thr**	141	mnv	gnomAD	**Lys316Arg**	316	Missence	gnomAD
**Trp226Ter**	226	Nonsence	PMID: 28236341	**Gly146Ala**	146	Missence	gnomAD	**Arg319Gln**	319	mnv	gnomAD
**c.801+1G>C**	267	splicing	PMID: 10664228	**His150Leu**	150	Missence	gnomAD	**Arg319Leu**	319	mnv	gnomAD
**Trp276Cys**	276	Missence	PMID: 16861859	**Ile151Val**	151	Missence	gnomAD	**Arg319Trp**	319	mnv	gnomAD
**Trp276Cys**	276	Missence	PMID: 12355085	**Ile153Thr**	153	Missence	gnomAD	**Ala322Val**	322	Missence	gnomAD
**Trp276Cys**	276	Missence	OMIM	**Asp154Gly**	154	Missence	gnomAD	**Val325Ile**	325	Missence	gnomAD
**Arg289IlefsTer9**	289	Frameshift	PMID: 25118007	**Ile157Val**	157	Missence	gnomAD	**Val331Leu**	331	Missence	gnomAD
**Phe292Leu**	292	Missence	PMID: 12189494	**c.483+1G>A**	161	splicing	gnomAD	**Ala333Val**	333	Missence	gnomAD
**Tyr293Ter**	293	Nonsence	PMID: 10664228	**Gly170Arg**	170	Missence	gnomAD	**Thr348Ser**	348	Missence	gnomAD
**Tyr293Ter**	293	Nonsence	PMID: 8852658	**Glu172Ala**	172	mnv	gnomAD	**Leu349Phe**	349	Missence	gnomAD
**Ala310Thr**	310	Missence	PMID: 10664228	**Glu172Asp**	172	mnv	gnomAD	**Asn351Asp**	351	mnv	gnomAD
**Arg319Trp**	319	Missence	PMID: 11471546	**Glu172Gln**	172	mnv	gnomAD	**Asn351Lys**	351	mnv	gnomAD
**Ile372AsnfsTer11**	372	Frameshift	PMID: 10664228	**Cys174LeufsTer17**	174	Frameshift	gnomAD	**Gln352Arg**	352	Missence	gnomAD
**Met374Ile**	374	Missence	PMID: 8630503	**Leu176Val**	176	Missence	gnomAD	**Asn353Lys**	353	Missence	gnomAD

**Met374Ile**	374	Missence	PMID: 28236341	**Val177Leu**	177	Missence	gnomAD	**Leu360His**	360	Missence	gnomAD
**Asn378Ile**	378	Missence	PMID: 8852659	**Pro178Thr**	178	Missence	gnomAD	**Ser362Arg**	362	Missence	gnomAD
**Pro383Leu**	383	Missence	PMID: 8852660	**Ile180Leu**	180	mnv	gnomAD	**c.1086+1T>A**	362	splicing	gnomAD
**Leu388TrpfsTer2**	388	Frameshift	PMID: 28236341	**Ile180Met**	180	mnv	gnomAD	**Leu364Val**	364	Missence	gnomAD
**Gly135Ser**	135	Missence	PMID: 35790984	**Ser191Cys**	191	Missence	gnomAD	**Tyr369Phe**	369	Missence	gnomAD
**Val185Met**	185	Missence	PMID: 35790984	**Leu192Val**	192	Missence	gnomAD	**Ile370Thr**	370	Missence	gnomAD
**Pro3Leu**	3	Missence	gnomAD	**Ala194Val**	194	Missence	gnomAD	**Gly371Ser**	371	Missence	gnomAD
**Pro4Ala**	4	Missence	gnomAD	**Arg201Gln**	201	mnv	gnomAD	**Leu377Val**	377	Missence	gnomAD
**Pro5Thr**	5	Missence	gnomAD	**Arg201Pro**	201	mnv	gnomAD	**Cys380Ser**	380	Missence	gnomAD
**Cys8Gly**	8	Missence	gnomAD	**Arg201Ter**	201	Nonsence	gnomAD	**Ile381Val**	381	Missence	gnomAD
**Ala11Val**	11	Missence	gnomAD	**Val203Ile**	203	mnv	gnomAD	**Ile384Val**	384	Missence	gnomAD
**Val16Phe**	16	Missence	gnomAD	**Val203Leu**	203	mnv	gnomAD	**Lys391Arg**	391	Missence	gnomAD
**Gly20Ser**	20	Missence	gnomAD	**Trp206Ter**	206	Nonsence	gnomAD	**Phe393Leu**	393	Missence	gnomAD
**Arg23Trp**	23	Missence	gnomAD	**Ser207Asn**	207	Missence	gnomAD	**Phe397Val**	397	Missence	gnomAD
**Gly26Arg**	26	Missence	gnomAD	**Arg208Lys**	208	Missence	gnomAD	**c.1194+1G>C**	398	splicing	gnomAD
**Gly30Asp**	30	Missence	gnomAD	**Gly211Val**	211	Missence	gnomAD	**Ser399Thr**	399	Missence	gnomAD
**Pro32Ser**	32	Missence	gnomAD	**Ile212Thr**	212	Missence	gnomAD	**Cys400Trp**	400	Missence	gnomAD
**Asp34Glu**	34	Missence	gnomAD	**Val214Ile**	214	Missence	gnomAD	**Trp404Ter**	404	Nonsence	gnomAD
**Arg35Thr**	35	Missence	gnomAD	**Trp217Leu**	217	Missence	gnomAD	**Ser407LeufsTer7**	407	Frameshift	gnomAD
**Ala36Asp**	36	Missence	gnomAD	**Leu224Ser**	224	Missence	gnomAD	**Phe408Leu**	408	Missence	gnomAD
**Thr37Ile**	37	Missence	gnomAD	**Ile225Thr**	225	Missence	gnomAD	**Ser419Leu**	419	Missence	gnomAD
**Pro38Leu**	38	Missence	gnomAD	**Val227Met**	227	Missence	gnomAD	**Lys422Asn**	422	mnv	gnomAD
**Ala43Ser**	43	Missence	gnomAD	**Val228Ala**	228	mnv	gnomAD	**Lys422Glu**	422	mnv	gnomAD
**Thr47Lys**	47	Missence	gnomAD	**Val228Phe**	228	mnv	gnomAD	**Lys424Glu**	424	Missence	gnomAD
**Leu53Ser**	53	Missence	gnomAD	**Ala233Gly**	233	Missence	gnomAD	**Ala425Pro**	425	Missence	gnomAD

**Trp54Arg**	54	Missence	gnomAD	**Ile238Thr**	238	mnv	gnomAD	**Asp427Gly**	427	Missence	gnomAD
**Pro55Arg**	55	Missence	gnomAD	**Ile238Val**	238	mnv	gnomAD	**His428Gln**	428	Missence	gnomAD
**Lys56Thr**	56	Missence	gnomAD	**Gly239Ser**	239	Missence	gnomAD	**Gly429Arg**	429	mnv	gnomAD
**Ala60Pro**	60	mnv	gnomAD	**Asp241Ala**	241	mnv	gnomAD	**Gly429Glu**	429	mnv	gnomAD
**Ala60Thr**	60	mnv	gnomAD	**Asp241Asn**	241	mnv	gnomAD	**Tyr430His**	430	Missence	gnomAD
**Arg64Gly**	64	mnv	gnomAD	**Met245Ile**	245	mnv	gnomAD	**Arg434Cys**	434	mnv	gnomAD
**Arg64Leu**	64	mnv	gnomAD	**Met245Lys**	245	mnv	gnomAD	**Arg434His**	434	mnv	gnomAD
**Arg64Pro**	64	mnv	gnomAD	**Asp246Asn**	246	mnv	gnomAD	**Arg434Ser**	434	mnv	gnomAD
**Arg64Trp**	64	mnv	gnomAD	**Asp246Gly**	246	mnv	gnomAD	**Ser435Tyr**	435	Missence	gnomAD
**Glu70Lys**	70	Missence	gnomAD	**Asp246Tyr**	246	mnv	gnomAD	**Ser436Gly**	436	mnv	gnomAD
**Pro72Ser**	72	Missence	gnomAD	**Asp246Val**	246	mnv	gnomAD	**Ser436Thr**	436	mnv	gnomAD
**Lys73Glu**	73	Missence	gnomAD	**Tyr247Cys**	247	Missence	gnomAD	**Lys438AsnfsTer27**	438	Frameshift	gnomAD
**Asp75Asn**	75	Missence	gnomAD	**Lys248Thr**	248	Missence	gnomAD	**Tyr439His**	439	Missence	gnomAD
**Arg76Met**	76	mnv	gnomAD	**Gly249Arg**	249	Missence	gnomAD	**Ser441Leu**	441	Missence	gnomAD
**Arg76Trp**	76	mnv	gnomAD	**Ser250Gly**	250	mnv	gnomAD				

Note: Pos, the position of amino acid (NP_000106.1); mnv, multi-nucleotide variants; gnomAD, the genome aggregation database, (https://gnomAD.broadinstitute.org/).

Table S5. Representative common variants in *EDNRB* associated with HSCR.

							Our data		Combined data			
**SNP**	**Chr**	**BP**	**A1**	**A2**	**MAF(Control)**	**MAF(Case)**	***OR***	***P***	***OR***	***P***	**R^2**	**Epigenomic Feature**
rs112706009	13	78509866	T	A	0.11	0.16	1.76 [1.40-2.20]	8.14×10^{-07}	1.74[1.46-2.07]	4.82×10^{-10}	1.00	H3K27me3 (HSMMtube); H3K4me1 (HUES6)
rs145150547	13	78588107	G	A	0.04	0.07	2.42 [1.65-3.55]	6.35×10^{-06}	2.22[1.70-2.89]	1.77×10^{-09}	0.20	H3K9me3 (H9 ENCSR323FKB); H3K27me3 (neural stem progenitor cell)
rs12720168	13	78491255	G	T	0.09	0.13	1.77 [1.38-2.26]	6.56×10^{-06}	1.77[1.46-2.15]	4.75×10^{-09}	0.71	H3K27me3 (bipolar neuron); H3K27ac (Fetal Intestine Large)

rs3027139	13	78490067	G	A	0.09	0.13	1.77 [1.38-2.26]	6.62×10^{-06}	1.74[1.44-2.11]	1.09×10^{-08}	0.71	H3K4me1 (fibroblast of dermis); H3K27ac (Fetal Intestine Large)
rs79823778	13	78430566	A	G	0.08	0.13	1.77 [1.37-2.27]	9.16×10^{-06}	1.71[1.41-2.08]	5.59×10^{-08}	0.64	H3K27me3 (A673); H3K4me1 (HUES64)
rs138218703	13	78429554	TG	T	0.08	0.13	1.76 [1.37-2.27]	9.83×10^{-06}	1.70[1.39-2.08]	1.72×10^{-07}	0.65	H3K4me1 (bipolar neuron); H3K4me1 (neural stem progenitor cell)
rs75777788	13	78429276	C	T	0.08	0.13	1.76 [1.37-2.27]	9.95×10^{-06}	1.71[1.40-2.07]	6.49×10^{-08}	0.65	H3K4me1 (bipolar neuron); H3K4me1 (endodermal cell)

In the table, SNPs with P value < 1×10^{-5} and LD R^2 < 0.8 are shown. The full list of SNPs with a P value < 1×10^{-3} can be found in **Supplementary Table 6**. SNP, Single Nucleotide Polymorphism; CHR, Chromosome; BP, Base pair of where the SNP is located; A1/A2 indicates the protective allele and risk allele to disease; MAF is the minor allele frequency in East Asian population; The calculation of odds ratio (OR) is also based on the risk allele of each SNP; The P value indicates the significance based on allelic association tests; R^2 is the LD

relation with rs112706009; Combined data: a common database consisting of the Genome Database of Born In Guangzhou Cohort study (GDBIG, http://gdbig.bigcs.com.cn/) and the Westlake BioBank for Chinese (WBBC, https://wbbc.westlake.edu.cn/) and PGG.Han2.0, https://www.biosino.org/pgghan2/index). 1,665 unrelated samples in GDBIG, 4,044 samples of south China, and 7250 South coast Han samples in PGG. Han were included, resulting in a combined cohort of 12,959 Southern Chinese individuals; Epigenomic features in reported cell types/tissue

Table S6. All associated SNPs with *P*< 1×10^{-3} in *EDNRB* in our cohort.

SNP	Chr	BP(hg19)	A1	A2	OR	P-value	R^2	MAF	Epigenomic Feature
**rs112706009**	13	78509866	T	A	1.76 [1.4-2.2]	8.14E-07	1.00	0.10	H3K27me3 (HSMMtube); H3K4me1 (HUES6)
**rs113837660**	13	78510351	A	G	1.76 [1.4-2.2]	8.16E-07	1.00	0.10	N/A
**rs77529095**	13	78570200	G	A	1.75 [1.4-2.18]	8.20E-07	0.96	0.10	H3K9me3 (bipolar neuron); H3K9me3 (HUES6)
**rs3027155**	13	78579823	G	A	1.75 [1.4-2.18]	8.23E-07	0.96	0.10	N/A
**rs111855642**	13	78513270	A	G	1.75 [1.4-2.19]	8.26E-07	1.00	0.10	H3K9me3 (bipolar neuron); H3K9me3 (HUES6)
**rs4366606**	13	78513841	C	T	1.75 [1.4-2.19]	8.27E-07	1.00	0.10	H3K9me3 (bipolar neuron); H3K79me2 (H1-trophoblast)
**rs12720146**	13	78518352	A	G	1.75 [1.4-2.19]	8.27E-07	1.00	0.10	H3K4me1 (H9 ENCSR323FKB); H3K9me3 (HUES64)
**rs78973758**	13	78518848	A	G	1.75 [1.4-2.19]	8.28E-07	1.00	0.10	H3K9me3 (HepG2); H3K4me1 (HUES64)
**rs75709517**	13	78589946	G	C	1.75 [1.4-2.18]	8.28E-07	0.96	0.10	H3K9me3 (HepG2); H3K27me3 (neural stem progenitor cell)
**rs79903738**	13	78595608	G	A	1.75 [1.4-2.18]	8.61E-07	0.96	0.10	H3K4me1 (bipolar neuron); H3K4me1 (neuron)
**rs148400335**	13	78507692	A	G	1.76 [1.4-2.2]	9.01E-07	0.99	0.10	Dnase1 (H9 ENCSR323FKB); H3K4me1 (HUES64)
**rs75513376**	13	78497040	T	G	1.75 [1.4-2.2]	9.43E-07	0.99	0.10	H3K27me3 (A673); H3K27me3 (common myeloid progenitor CD34 positive ENCSR337XXD 1)
**rs78320535**	13	78559817	G	A	1.75 [1.4-2.18]	1.01E-06	0.98	0.10	N/A
**rs74517527**	13	78556003	C	T	1.75 [1.4-2.18]	1.01E-06	0.98	0.10	H3K4me1 (skeletal muscle myoblast); H2BK12ac (IMR 90)
**rs79796321**	13	78554303	A	T	1.75 [1.4-2.18]	1.01E-06	0.98	0.10	H3K4me1 (skeletal muscle myoblast); H3K4me1 (endodermal cell)
**rs149080132**	13	78536088	C	G	1.75 [1.4-2.18]	1.01E-06	0.99	0.10	H3K9me3 (A673); H3K9me3 (common myeloid progenitor CD34 positive ENCSR337XXD 1)
**rs8000027**	13	78537565	C	G	1.75 [1.4-2.18]	1.01E-06	0.99	0.10	H3K9me3 (bipolar neuron); H3K9me3 (common myeloid progenitor CD34 positive ENCSR337XXD 1)

**rs113137166**	13	78535124	G	A	1.74 [1.4-2.18]	1.02E-06	0.99	0.10	H3K9me3 (HepG2); H3K9me3 (HUES6)
**rs11838546**	13	78531614	G	A	1.74 [1.4-2.18]	1.02E-06	0.99	0.10	H3K9me3 (HepG2); H3K9me3 (HUES6)
**rs74240503**	13	78530574	A	G	1.74 [1.4-2.18]	1.03E-06	0.99	0.10	H3K9me3 (HepG2); H3K9me3 (endodermal cell)
**rs7998775**	13	78522427	C	T	1.74 [1.4-2.18]	1.03E-06	0.99	0.10	N/A
**rs78914345**	13	78564109	G	C	1.74 [1.39-2.18]	1.03E-06	0.98	0.10	H3K27me3 (DND-41); H3K4me1 (lung ENCSR465WKM)
**rs12720138**	13	78526587	C	T	1.74 [1.4-2.18]	1.03E-06	0.99	0.10	H3K27me3 (bipolar neuron); H3K4me1 (Placenta)
**rs149902099**	13	78552154	A	G	1.74 [1.39-2.18]	1.08E-06	0.98	0.10	H3K36me3 (bipolar neuron); H3K9me3 (endodermal cell)
**rs78789229**	13	78505836	A	T	1.72 [1.38-2.15]	1.83E-06	0.97	0.10	H3K27me3 (A673); H3K4me1 (HUES64)
**rs9544636**	13	78499569	C	T	1.72 [1.38-2.15]	1.83E-06	0.97	0.10	H3K27me3 (A673); H3K27me3 (IMR 90)
**rs7323156**	13	78499219	A	C	1.72 [1.38-2.15]	1.85E-06	0.97	0.10	H3K9me3 (HepG2); H3K9me3 (HUES6)
**rs9544635**	13	78498079	T	C	1.72 [1.38-2.15]	1.89E-06	0.97	0.10	N/A
**rs7997107**	13	78497427	C	G	1.72 [1.38-2.15]	1.89E-06	0.97	0.10	N/A
**rs145150547**	13	78588107	G	A	2.42 [1.65-3.55]	6.35E-06	0.20	0.02	H3K9me3 (H9 ENCSR323FKB); H3K27me3 (neural stem progenitor cell)
**rs12720168**	13	78491255	G	T	1.77 [1.38-2.26]	6.56E-06	0.71	0.07	H3K27me3 (bipolar neuron); H3K27ac (Fetal Intestine Large)
**rs3027139**	13	78490067	G	A	1.77 [1.38-2.26]	6.62E-06	0.71	0.07	H3K4me1 (fibroblast of dermis); H3K27ac (Fetal Intestine Large)
**rs79823778**	13	78430566	A	G	1.77 [1.37-2.27]	9.16E-06	0.64	0.07	H3K27me3 (A673); H3K4me1 (HUES64)
**rs138218703**	13	78429554	TG	T	1.76 [1.37-2.27]	9.83E-06	0.65	0.07	H3K4me1 (bipolar neuron); H3K4me1 (neural stem progenitor cell)
**rs75777788**	13	78429276	C	T	1.76 [1.37-2.27]	9.95E-06	0.65	0.07	H3K4me1 (bipolar neuron); H3K4me1 (endodermal cell)
**rs75150877**	13	78426216	T	G	1.76 [1.37-2.26]	1.10E-05	0.59	0.07	H3K9me3 (HepG2); H3K9me3 (HUES6)
**rs12720207**	13	78468112	G	A	1.74 [1.36-2.24]	1.21E-05	0.65	0.07	H3K9me3 (Osteobl); H3K4me1 (HUES6)
**rs7993770**	13	78594904	T	G	0.62 [0.51-0.77]	1.31E-05	0.85	0.89	H3K4me1 (bipolar neuron); H3K4me1 (neuron)

**rs944382**	13	78595990	A	G	0.62 [0.51-0.77]	1.31E-05	0.85	0.89	H3K4me1 (bipolar neuron); H3K4me1 (neuron)
**rs9544673**	13	78593728	A	T	0.63 [0.51-0.77]	1.38E-05	0.85	0.89	H3K9me3 (HepG2); H3K27me3 (neural stem progenitor cell)
**rs4332645**	13	78594589	T	C	0.63 [0.51-0.77]	1.48E-05	0.84	0.89	H3K9me3 (HepG2); H3K36me3 (Right Atrium)
**rs9593265**	13	78456638	C	A	1.73 [1.35-2.22]	1.66E-05	0.66	0.07	N/A
**rs2147555**	13	78479385	A	C	1.71 [1.34-2.18]	1.77E-05	0.67	0.08	N/A
**rs9530711**	13	78584056	A	G	0.63 [0.51-0.78]	1.82E-05	0.85	0.89	H3K9me3 (HepG2); H3K27me3 (neural stem progenitor cell)
**rs9600946**	13	78464096	G	A	1.72 [1.34-2.21]	1.86E-05	0.66	0.07	N/A
**rs3027082**	13	78427952	T	A	1.72 [1.34-2.21]	1.87E-05	0.63	0.07	H3K4me1 (bipolar neuron); H3K4me1 (HUES6)
**rs1572091**	13	78428918	G	T	1.72 [1.34-2.21]	1.88E-05	0.63	0.07	H3K4me1 (bipolar neuron); H3K4me1 (endodermal cell)
**rs74685263**	13	78460856	T	A	1.72 [1.34-2.21]	1.88E-05	0.66	0.07	N/A
**rs1819436**	13	78580283	T	C	0.63 [0.51-0.78]	1.98E-05	0.85	0.89	H3K9me3 (HepG2); H3K9me3 (Fetal Adrenal Gland)
**rs2329040**	13	78436486	A	C	1.7 [1.33-2.17]	2.49E-05	0.63	0.07	H3K4me1 (A673); H3K4me1 (lung ENCSR465WKM)
**rs9600943**	13	78442587	C	T	1.69 [1.32-2.17]	2.62E-05	0.63	0.07	H3K9me3 (CD14 positive monocyte); H2BK12ac (IMR 90)
**rs2296281**	13	78474490	C	G	1.68 [1.32-2.15]	2.88E-05	0.66	0.08	H3K27me3 (HCT116); H3K36me3 (HUES64)
**rs76629678**	13	78521666	C	G	1.78 [1.35-2.34]	3.41E-05	0.62	0.06	N/A
**rs9544627**	13	78461008	G	A	1.68 [1.31-2.14]	3.89E-05	0.64	0.07	H3K27me3 (myotube); H3K79me1 (H1-trophoblast)
**rs141469258**	13	78422344	AAAAT	A	0.61 [0.49-0.77]	4.12E-05	0.42	0.90	H3K9me3 (HUES64)
**rs117726541**	13	78586242	C	T	0.44 [0.28-0.7]	0.000515	0.00	0.04	H3K9me3 (HepG2); H3K27me3 (neural stem progenitor cell)
**rs118084964**	13	78503955	G	A	0.42 [0.25-0.69]	0.000608	0.00	0.03	H3K4me1 (Monocytes-CD14+); H3K4me1 (Monocytes-CD14+ (PB) Roadmap)
**rs187817356**	13	78507958	A	G	0.42 [0.26-0.69]	0.000672	0.00	0.03	H3K27me3 (SK N SH); H3K4me1 (HUES64)
**rs192906585**	13	78508576	A	G	0.42 [0.26-0.69]	0.000677	0.00	0.03	H3K4me1 (HeLa-S3); H3K4me1 (HUES64)

**rs143301709**	13	78508956	A	G	0.42 [0.26-0.69]	0.00068	0.00	0.03	H3K27me3 (SK N SH); H3K4me1 (HUES6)
**rs148235670**	13	78508981	T	C	0.42 [0.26-0.69]	0.00068	0.00	0.03	H3K4me1 (bipolar neuron); H3K4me1 (HUES6)
**rs190915272**	13	78509362	A	G	0.42 [0.26-0.69]	0.000684	0.00	0.03	N/A
**rs144501827**	13	78509738	T	C	0.42 [0.26-0.69]	0.000686	0.00	0.03	H3K27me3 (HSMMtube); H3K4me1 (HUES6)
**rs148768775**	13	78509865	T	G	0.42 [0.26-0.69]	0.000686	0.00	0.03	H3K27me3 (HSMMtube); H3K4me1 (HUES6)
**rs145677776**	13	78510053	A	G	0.42 [0.26-0.69]	0.000691	0.00	0.03	H3K27me3 (HSMMtube); H3K9ac (HUES6)
**rs189498422**	13	78510584	C	T	0.42 [0.26-0.7]	0.000696	0.00	0.03	H3K27me3 (A673); H3K9ac (HUES6)
**rs146030855**	13	78510818	C	T	0.42 [0.26-0.7]	0.000698	0.00	0.03	H3K27me3 (A673); H3K9ac (HUES6)
**rs141173102**	13	78511075	T	C	0.42 [0.26-0.7]	0.0007	0.00	0.03	H3K27me3 (A673); H3K9ac (HUES6)
**rs140108197**	13	78511401	C	G	0.42 [0.26-0.7]	0.000704	0.00	0.03	H3K27me3 (A673); H3K9ac (HUES6)
**rs145612709**	13	78511712	T	C	0.42 [0.26-0.7]	0.000706	0.00	0.03	H3K27me3 (A673); H3K9ac (HUES6)
**rs200434699**	13	78511867	GA	G	0.42 [0.26-0.7]	0.00070774	0.00	0.03	H3K9me3 (bipolar neuron); H3K9ac (HUES6)
**rs147755646**	13	78511857	A	G	0.42 [0.26-0.7]	0.000708	0.00	0.03	H3K27me3 (A673); H3K9ac (HUES6)
**rs148716061**	13	78511891	C	T	0.42 [0.26-0.7]	0.000709	0.00	0.03	H3K9me3 (bipolar neuron); H3K9ac (HUES6)
**rs117654536**	13	78512077	T	C	0.42 [0.26-0.7]	0.00071	0.00	0.03	H3K9me3 (bipolar neuron); H3K9ac (HUES6)
**rs117151827**	13	78512451	G	C	0.42 [0.26-0.7]	0.000713	0.00	0.03	H3K9me3 (bipolar neuron); H3K9ac (HUES6)
**rs190085355**	13	78512785	A	G	0.42 [0.26-0.7]	0.000715	0.00	0.03	H3K9me3 (bipolar neuron); H3K9ac (HUES6)
**rs149240017**	13	78513189	G	C	0.42 [0.26-0.7]	0.000718	0.00	0.03	H3K9me3 (bipolar neuron); H3K9ac (HUES6)
**rs141667464**	13	78513349	G	C	0.42 [0.26-0.7]	0.000721	0.00	0.03	H3K9me3 (bipolar neuron); H3K9me3 (HUES6)
**rs140022061**	13	78513941	G	A	0.42 [0.26-0.7]	0.000723	0.00	0.03	H3K9me3 (bipolar neuron); H3K9me3 (HUES6)
**rs112588208**	13	78513487	G	A	0.42 [0.26-0.7]	0.000724	0.00	0.03	H3K9me3 (bipolar neuron); H2BK12ac (H1 hESC)
**rs144041382**	13	78513888	T	A	0.42 [0.26-0.7]	0.000724	0.00	0.03	H3K27me3 (HCT116); H3K9me3 (HUES6)
**rs183920853**	13	78514062	T	C	0.42 [0.26-0.7]	0.000725	0.00	0.03	N/A
**rs188174307**	13	78514063	G	A	0.42 [0.26-0.7]	0.000725	0.00	0.03	N/A
**rs145693393**	13	78514182	G	A	0.42 [0.26-0.7]	0.000725	0.00	0.03	N/A

**rs118083708**	13	78514277	G	A	0.42 [0.26-0.7]	0.000725	0.00	0.03	N/A
**rs201464166**	13	78514661	TA	T	0.42 [0.26-0.7]	0.00072617	0.00	0.03	H3K9me3 (HepG2); H3K9me3 (endodermal cell)
**rs117481328**	13	78515879	T	C	0.42 [0.26-0.7]	0.000732	0.00	0.03	H3K9me3 (HepG2); H3K4me1 (lung ENCSR465WKM)
**rs118121837**	13	78516146	A	C	0.42 [0.26-0.7]	0.000732	0.00	0.03	H3K27me3 (HSMMtube); H3K36me3 (H1-trophoblast)
**rs117061453**	13	78516196	T	C	0.42 [0.26-0.7]	0.000732	0.00	0.03	H3K27me3 (HSMMtube); H3K36me3 (H1-trophoblast)
**rs79061008**	13	78516956	G	A	0.42 [0.26-0.7]	0.000734	0.00	0.03	N/A
**rs117079001**	13	78516978	T	C	0.42 [0.26-0.7]	0.000734	0.00	0.03	N/A
**rs12720150**	13	78517277	A	C	0.42 [0.26-0.7]	0.000735	0.00	0.03	H3K27me3 (myotube); H3K36me3 (HUES64)
**rs12720147**	13	78518218	G	T	0.43 [0.26-0.7]	0.000737	0.00	0.03	H3K9me3 (bipolar neuron); H3K9me3 (HUES64)
**rs145551307**	13	78519158	G	A	0.43 [0.26-0.7]	0.000737	0.00	0.03	H3K9me3 (HepG2); H3K4me1 (HUES64)
**rs117616227**	13	78519205	C	T	0.43 [0.26-0.7]	0.000737	0.00	0.03	H3K9me3 (HepG2); H3K4me1 (HUES64)
**rs117859695**	13	78519283	T	A	0.43 [0.26-0.7]	0.000737	0.00	0.03	H3K9me3 (HepG2); H3K4me1 (HUES64)
**rs117204143**	13	78519332	T	A	0.43 [0.26-0.7]	0.000738	0.00	0.03	H3K27me3 (HSMMtube); H3K4me1 (HUES64)
**rs76552180**	13	78519742	A	C	0.43 [0.26-0.7]	0.000738	0.00	0.03	N/A
**rs138864503**	13	78520259	G	A	0.43 [0.26-0.7]	0.000738	0.00	0.03	H3K27me3 (myotube); H3K4me1 (HUES64)
**rs117541814**	13	78435442	G	A	0.44 [0.27-0.71]	0.000754	0.00	0.04	H3K4me1 (A673); H3K4me1 (lung ENCSR465WKM)
**rs148925967**	13	78482264	T	C	0.43 [0.27-0.71]	0.000782	0.00	0.04	Dnase1 (HUVEC); H3K4me1 (Fetal Intestine Small)
**rs117697898**	13	78489404	C	T	0.43 [0.27-0.71]	0.000789	0.00	0.04	H3K27me3 (bipolar neuron); H3K27ac (Fetal Intestine Large)
**rs12720173**	13	78491028	C	T	0.43 [0.27-0.71]	0.000789	0.00	0.04	H3K27me3 (bipolar neuron); H3K27ac (Fetal Intestine Large)
**rs12720176**	13	78490910	C	A	0.43 [0.27-0.71]	0.00079	0.00	0.04	H3K27me3 (bipolar neuron); H3K4me1 (HUES48)
**rs12720158**	13	78493219	A	C	0.44 [0.27-0.71]	0.000814	0.00	0.04	H3K27me3 (bipolar neuron); H3K27ac (endodermal cell)
**rs76463081**	13	78466360	G	C	0.44 [0.27-0.71]	0.000894	0.00	0.04	H3K4me1 (bipolar neuron); H3K4me1 (Monocytes-CD14+ (PB) Roadmap)

**rs117743239**	13	78467665	G	A	0.44 [0.27-0.71]	0.000894	0.00	0.04	H3K4me1 (bipolar neuron); H3K4me1 (HUES6)
**rs143224600**	13	78465829	G	A	0.44 [0.27-0.71]	0.000895	0.00	0.04	H3K4me1 (bipolar neuron); H3K4me1 (Monocytes-CD14+ (PB) Roadmap)
**rs3027092**	13	78470568	G	A	0.44 [0.27-0.71]	0.000895	0.00	0.04	H3K4me1 (bipolar neuron); H3K27ac (Fetal Adrenal Gland)
**rs138377414**	13	78463755	A	C	0.44 [0.27-0.71]	0.000896	0.00	0.04	N/A
**rs117859377**	13	78465064	G	A	0.44 [0.27-0.71]	0.000896	0.00	0.04	H3K4me1 (bipolar neuron); H3K4me1 (Monocytes-CD14+ (PB) Roadmap)
**rs201074913**	13	78452807	ATTAT	A	0.44 [0.27-0.71]	0.00093325	0.00	0.04	H3K4me1 (fibroblast of dermis); H4K8ac (IMR 90)
**rs199730897**	13	78449089	CT	C	0.44 [0.27-0.71]	0.00094588	0.00	0.04	H3K4me1 (NHDF-AD); H4K8ac (IMR 90)
**rs75097976**	13	78442141	A	C	0.44 [0.27-0.72]	0.000969	0.00	0.04	H3K9me3 (CD14 positive monocyte); H2BK12ac (IMR 90)
**rs146260345**	13	78440307	A	G	0.44 [0.27-0.72]	0.000977	0.00	0.04	H3K9me3 (HepG2); H2AK5ac (IMR90)
**rs80209843**	13	78437565	A	G	0.44 [0.27-0.72]	0.000982	0.00	0.04	H3K4me1 (A673); H3K4me1 (Monocytes-CD14+ (PB) Roadmap)
**rs79194883**	13	78436882	A	G	0.44 [0.27-0.72]	0.000985	0.00	0.04	H3K4me1 (A673); H3K4me1 (Monocytes-CD14+ (PB) Roadmap)

Note: SNP, Single Nucleotide Polymorphism; CHR, Chromosome; BP, Base pair of where the SNP is located; A1/A2 indicates the protective allele and risk allele to disease; MAF is the minor allele frequency in our cohort (a Southern Chinese cohort comprising 454 HSCR cases and 2075 controls); The P value indicates the significance based on allelic association tests; The calculation of odds ratio (OR) is also based on the risk allele of each SNP;N/A: Not available; R^2 is the LD relation with rs112706009; Epigenomic features in reported cell types/tissues.

Table S7. Clinical information of subjects being sequenced in the HSCR family.

ID	Sex	Age (year)	Ethnicity	Diagnosis	Clinical and laboratory manifestations
I-1	F	57	Southern Chinese	Healthy	Healthy
II-2	M	25	Southern Chinese	HSCR	Self-reported Hirschsprung's disease
II-1	F	23	Southern Chinese	Healthy	Healthy
III-1	M	11	Southern Chinese	HSCR	Presented with a history of Hirschsprung's disease
III-2 (HD7) (proband)	M	0.5	Southern Chinese	L-HSCR	Presented with vomit, distension, and failure to pass meconium. The birth weight was 3.15 kg. Colon biopsy at day 3 after birth demonstrated that proximal rectum was aganglionic, transverse colon and ileum had ganglion cells. Ileostomy was performed. She was readmitted to the hospital at the 6th month due to expulsion delay in the being enema test. The radical surgery was then performed.

Note: F:female; M:male; L-HSCR: long-segmental HSCR.
